# Roles Nrf2 Plays in Myeloid Cells and Related Disorders

**DOI:** 10.1155/2013/529219

**Published:** 2013-06-02

**Authors:** Eri Kobayashi, Takafumi Suzuki, Masayuki Yamamoto

**Affiliations:** Department of Medical Biochemistry, Tohoku University Graduate School of Medicine, 2-1 Seiryo-machi, Aoba-ku, Sendai, Miyagi 980-8575, Japan

## Abstract

The Keap1-Nrf2 system protects animals from oxidative and electrophilic stresses. Nrf2 is a transcription factor that induces the expression of genes essential for detoxifying reactive oxygen species (ROS) and cytotoxic electrophiles. Keap1 is a stress sensor protein that binds to and ubiquitinates Nrf2 under unstressed conditions, leading to the rapid proteasomal degradation of Nrf2. Upon exposure to stress, Keap1 is modified and inactivated, which allows Nrf2 to accumulate and activate the transcription of a battery of cytoprotective genes. Antioxidative and detoxification activities are important for many types of cells to avoid DNA damage and cell death. Accumulating lines of recent evidence suggest that Nrf2 is also required for the primary functions of myeloid cells, which include phagocytosis, inflammation regulation, and ROS generation for bactericidal activities. In fact, results from several mouse models have shown that Nrf2 expression in myeloid cells is required for the proper regulation of inflammation, antitumor immunity, and atherosclerosis. Moreover, several molecules generated upon inflammation activate Nrf2. Although ROS detoxification mediated by Nrf2 is assumed to be required for anti-inflammation, the entire picture of the Nrf2-mediated regulation of myeloid cell primary functions has yet to be elucidated. In this review, we describe the Nrf2 inducers characteristic of myeloid cells and the contributions of Nrf2 to diseases.

## 1. Introduction

**Figure 1:**
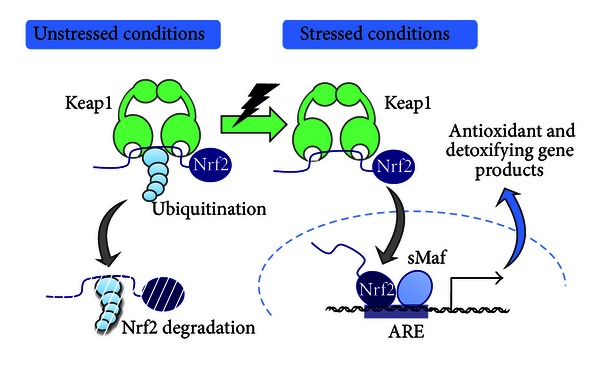
Keap1-Nrf2 stress response system. Stress-sensing system of Keap1 and Nrf2. Environmental stresses, including ROS and electrophiles, inactivate Keap1 and stall the ubiquitination and degradation of Nrf2. Nrf2 accumulates in the nucleus and forms a heterodimer with the sMaf protein. The binding of the Nrf2-sMaf heterodimer to the EpRE/ARE motif leads to the transactivation of Nrf2 target genes, which include a battery of antioxidant and detoxifying genes required for cellular protection.

NF-E2-related factor like-2 (Nrf2) is a transcription factor that activates a battery of genes that protect cells from reactive oxygen species (ROS) or toxic electrophiles [1, 2] (Figure 1). Nrf2 activity is strictly regulated through the stress sensor protein Keap1 (Kelch-like ECH-associated protein 1). Under unstressed conditions, Nrf2 is captured by Keap1 in the cytosol and is constitutively ubiquitinated and degraded by the proteasome [3–5]. By contrast, under stressed conditions, Keap1 senses stress or environmental insults and stops the degradation of Nrf2, resulting in the accumulation and nuclear translocation of the Nrf2 protein [6]. In the nucleus, Nrf2 dimerizes with small Maf proteins (sMaf), and the Nrf2-sMaf heterodimer binds to antioxidant/electrophile responsive elements (AREs/EpREs) to activate the expression of target genes [7, 8].

The chemicals that activate Nrf2 and Nrf2 inducers are structurally diverse but share a common electrophilic nature [9]. Of note, these inducers interact with certain reactive cysteine residues of Keap1 [10], which contains 25 cysteine residues [11]. This electrophilic modification results in the inhibition of the ubiquitin ligase activity of Keap1 [5, 12]. Typical Nrf2 inducers include diethyl maleate (DEM), tert-butylhydroquinone (tBHQ), sulforaphane (SFN), and 2-cyano-3,12-dioxooleana-1,9(11)-dien-28-oic acid (CDDO) derivatives [13]. In addition, upon the development of inflammation, several Nrf2-activating molecules accumulate, including 15-deoxy-Δ^12,14^-prostaglandin J_2_ (15d-PGJ_2_) [14], nitric oxide (NO), and NO-derived products [15–20]. In the following chapter, we will focus on how the Keap1-Nrf2 system responds to inflammatory signals in myeloid cells.

In initial analyses, Nrf2 was found to regulate the expression of many antioxidant and detoxifying enzymes and proteins [1, 21, 22]. For example, genes encoding glutathione *S*-transferases (GSTs) and NAD(P)H:quinone oxidoreductase 1 (*Nqo1*) are the prime targets of Nrf2 regulation [23]. Glutathione peroxidase 2 (*Gpx2*), glutamate cysteine lyase catalytic and regulatory subunits, and heme oxigenase-1 (*HO-1*) are also target genes of Nrf2 [24–26]. This list of Nrf2 target genes reveals that Nrf2 is critical for the maintenance of redox homeostasis within cells. In fact, Nrf2 deficiency in mice leads to oxidative stress conditions that cause DNA damage and cell death [27, 28].

Additionally, recent analyses have revealed that Nrf2 also regulates genes that are essential for cellular metabolism, cell proliferation, selective protein degradation, and immune response [29–32]. Regarding primary myeloid cell functions, inflammatory regulation and phagocytosis are also associated with the Keap1-Nrf2 pathway.

## 2. Nrf2 Activation Mediated by Inflammation-Related Molecules

**Figure 2:**
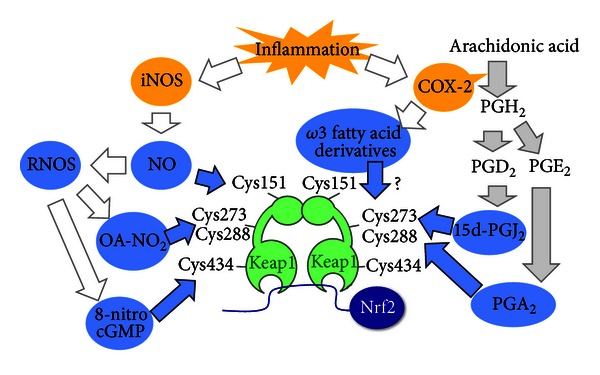
Nrf2 activation by inflammation-related molecules. Inflammation stimulates the production/accumulation of Nrf2-inducing molecules. Nrf2 inducers modify target reactive cysteine residues of Keap1; blue arrows indicate the flow of various Nrf2 inducers toward the reactive cysteine residues of Keap1. Inflammation induces COX-2, which leads to the production of 15d-PGJ_2_ and PGA_2_. COX-2 also produces electrophilic derivatives from *ω*3-fatty acids. PGA_2_, 15d-PGJ_2_, and *ω*3-fatty acid derivatives inactivate Keap1, leading to Nrf2 activation. Inflammation also induces iNOS and causes NO production. NO and its derivatives 8-nitro-cGMP and OA-NO_2_ also modify Keap1 and activate Nrf2. PGA_2_, 15d-PGJ_2_, *ω*3-fatty acid derivatives, and OA-NO_2_ preferentially modify Cys273/288 of Keap1, whereas NO preferentially modifies Cys151, and 8-nitro-cGMP preferentially modifies Cys434.

Two prevalent inflammatory signaling cascades, that is, the cyclooxygenase (COX)-2 pathway and the NO synthesis pathway, generate Nrf2-activating molecules (Figure 2). COX-2 catalyzes arachidonic acids and produces various bioactive prostaglandins. One of the COX-2 pathway products is 15d-PGJ_2_. Importantly, 15d-PGJ_2_ binds directly to cysteine residues of Keap1. 15d-PGJ_2_ is primarily produced by macrophages for inflammation resolution; thus, abrogating 15d-PGJ_2_ production with COX-2 inhibitors causes persistent neutrophil infiltration in carrageenan-induced pleurisy [14].

Although one type of Nrf2 inducers, including DEM, tBHQ, and SFN, modify cysteine residue 151 (Cys151) of Keap1, 15d-PGJ_2_ interacts with cysteine residues 273/288 (Cys273/288) of Keap1 [33, 34]. PGA_2_, another prostaglandin, also activates Nrf2 by binding to Cys273/288 [33]. In addition to these prostaglandins, COX-2 produces electrophilic *ω*3-fatty acid derivatives from eicosapentaenoic acids and docosahexaenoic acids, which induces Nrf2 and expression of its target genes in macrophages [35].

Although it is well accepted that NO activates Nrf2 by modifying Keap1, it remains to be clarified whether NO modifies Keap1 directly or through the generation of reactive nitrogen oxide species (RNOS). Consistent with the direct modification hypothesis, NO S-nitrosylates Cys151 of Keap1 [15, 16]. By contrast, the indirect hypothesis is supported by the observation that NO generates RNOS that nitrosylate cGMP and produce 8-nitroguanosine 3′,5′-cyclic monophosphate (8-nitro-cGMP) [17]. S-guanylation of Keap1 at Cys434 by 8-nitro-cGMP abrogates the Keap1-mediated inhibition of Nrf2 [18]. Alternatively, nitro fatty acids (OA-NO_2_) are produced by RNOS through the nitration of unsaturated fatty acids. OA-NO_2_ modifies Keap1 cysteines, primarily Cys273/288 [19, 20]. Thus, the Keap1-Nrf2 system appears to respond to multi-way signaling mechanisms utilizing NO.

## 3. Inflammatory Regulation by Nrf2 in Myeloid Cells

**Figure 3:**
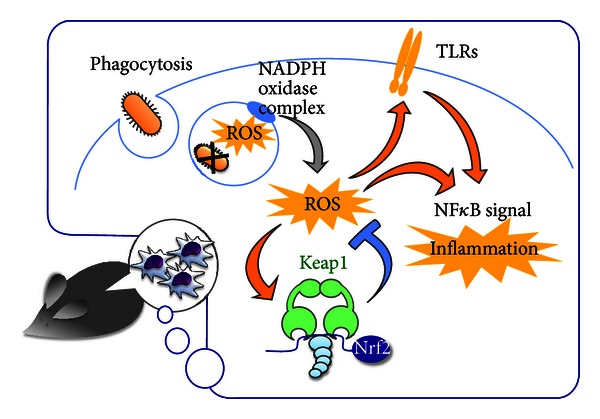
Suppression of acute inflammation by Nrf2. Nrf2 function related to acute inflammation in myeloid cells. The blue line represents the consequence of Nrf2 activation, that is, the loss of ROS, while the consequences of ROS hyperaccumulation are shown with orange arrows. Although ROS exacerbate inflammation, Nrf2 exerts anti-inflammatory effects through the suppression of ROS accumulation. In macrophages, ROS is primarily generated by the NADPH oxidase complex for bacterial killing.

Nrf2 deficiency in myeloid cells provokes ROS accumulation, as is the case for other cell lineages. Excessive ROS affect inflammatory regulation in myeloid cells (Figure 3), as ROS activate various inflammatory signaling pathways. One such pathway is the NF*κ*B (nuclear factor kappa B) pathway, the most potent activator of innate immunity, which induces the expression of various proinflammatory cytokines [36, 37]. ROS also enhance the translocation of TLRs (Toll-like receptors) to lipid rafts, in which signaling molecules cluster to effectively activate downstream signals [38, 39]. TLR accumulation in lipid rafts enhances inflammatory signals through the NF*κ*B and IRF3 (interferon regulatory transcription factor 3) pathways [39]. Consistently, macrophages from Nrf2-deficient mice show an increase in the LPS-induced activation of TLRs and NF*κ*B signaling, leading to an elevated expression of proinflammatory cytokines [40].

Intriguingly, the origins of ROS appear to differ between myeloid cells and other cell lineages. In macrophages and neutrophils, ROS are generated by the NADPH oxidase complex. ROS accumulate in phagosomes to kill engulfed pathogens but partially leak into the cytoplasm. Inherited defects in NADPH oxidase genes cause chronic granulomatous disease, which leads to life-threatening recurrent infections, demonstrating the importance of ROS in the bactericidal activity of phagocytes. Because the NADPH oxidase complex is the major source of ROS that accumulate in Nrf2-deficient myeloid cells, ROS accumulation in Nrf2-deficient phagocytes is abolished by the simultaneous knockout of the *Gp*91^*phox*^ gene, which encodes one component of the NADPH oxidase complex [40]. This ROS-generating feature of myeloid cells is in clear contrast to other general cells, in which mitochondria are the most potent source or generator of ROS.

Nrf2 is suggested to protect myeloid cells from excessive ROS generated during the immune response. Nrf2 also directly regulates the expression of inflammation-associated genes. For example, NRF2 activates *ATF3* (activating transcription factor 3) gene expression by binding to AREs in its promoter [32]. Because ATF3 represses the expression of the proinflammatory cytokine interleukin *(Il)6*, this NRF2-dependent activation of ATF3 exerts anti-inflammatory effects.

## 4. Nrf2 Regulates Phagocytosis

Phagocytosis is one of the myeloid-specific functions regulated by Nrf2. Nrf2-deficient myeloid cells show a decrease in phagocytosis and bactericidal activity, whereas Keap1-deficient myeloid cells in which Nrf2 is activated show an increase in these functions [41]. The decrease in phagocytosis in Nrf2-deficient mice is attributable to the absence of the LPS-induced expression of the scavenger receptor Marco, whose expression is also regulated by Nrf2 [42].

In this regard, it is interesting to note that Nrf2 regulates the differentiation of various types of cells. In 3T3-L1 cells, Nrf2 induces *Cebp*β** gene expression by binding to an ARE in its upstream promoter region and activating adipogenesis [43]. Because C/EBP*β* also regulates the differentiation of myeloid cells [44], we hypothesize that the Nrf2-C/EBP*β* axis may contribute to myeloid lineage differentiation.

## 5. Nrf2 and Acute Inflammation

Nrf2 expression in myeloid cells is tightly associated with a wide range of inflammation-related diseases. Of note, the Nrf2 contribution to myeloid cells is well known in a number of acute inflammation models, in which Nrf2 suppresses inflammation. For example, in lung inflammation models, Nrf2-deficient mice display more severe lung inflammation induced by cigarette smoke [45] and hyperoxia [46, 47] than wild-type mice, resulting in delayed recovery from emphysema. Nrf2-deficient mice also show worsened pneumonia caused by *Staphylococcus aureus* infection [48]. The antigen-specific immune response induced by sensitization to ovalbumin in a well-recognized asthma model is also aggravated by Nrf2 deficiency [49].

In addition to lung injury models, experimental sepsis has been exploited for the study of the Nrf2 contribution to acute inflammation. In Nrf2-deficient mice, sepsis caused by cecal ligation and puncture (CLP) gives rise to increased mortality compared with wild-type mice [50]. Endotoxin shock induced by the injection of a lethal dose of LPS leads to similar results, supporting the hypothesis that increased mortality in Nrf2-deficient mice is due to a hyper-activated inflammatory response but not a deficiency in bacterial killing ability. Because pretreatment with an antioxidant, N-acetylcysteine (NAC), improves the survival of Nrf2-deficient and wild-type mice after LPS-induced sepsis, the exacerbated inflammation in Nrf2-deficient mice appears to be attributable to excess ROS [50].

In these acute inflammation models, Nrf2 expression in myeloid cells is required for anti-inflammation. In bone marrow transplantation assays, recipient mice transplanted with Nrf2-deficient bone marrow cells display exacerbated porcine pancreatic elastase-induced emphysema similar to conventional Nrf2-deficient mice, although they have wild-type epithelial cells [51]. Similarly, the myeloid-specific deletion of Nrf2 leads to increased sepsis severity, whereas the myeloid-specific activation of Nrf2 through Keap1 conditional deletion leads to alleviated septic inflammation [41]. These observations clearly show that Nrf2 is an important regulator of acute inflammation in myeloid cells. Regarding the molecular basis, the Nrf2-mediated elimination of ROS seems to contribute to this process.

## 6. Antitumor Immunity and Nrf2

**Figure 4:**
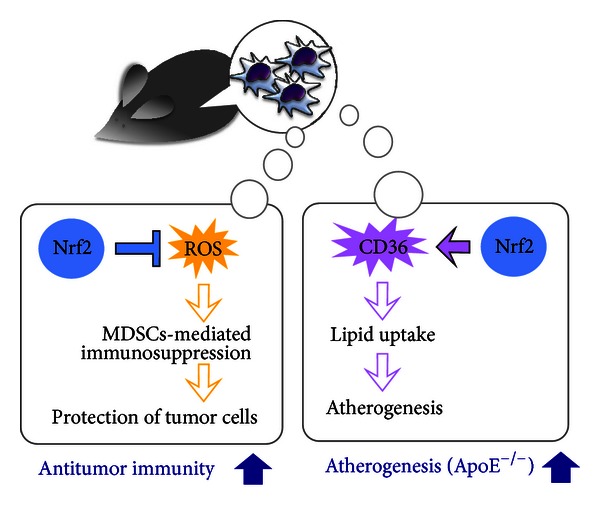
Physiological roles of Nrf2 in myeloid cells. The roles of Nrf2 in myeloid cells have been clarified in disease models. In tumor development models, Nrf2 suppresses ROS in MDSCs, in which ROS is required for the suppression of T cells. Thus, Nrf2 supports antitumor immunity and reduces tumor metastasis. In an ApoE-deficient atherosclerotic mouse model, Nrf2 upregulates the scavenger receptor CD36 and promotes atherogenesis.

As shown in Figure 4, one of the most intriguing findings in recent Nrf2 analyses is that Nrf2 supports antitumor immunity [52]. In the absence of Nrf2, tumor-supporting Gr1^+^CD11b^+^ cells, designated as myeloid-derived suppressor cells (MDSCs) [53], show a higher activity to attenuate the T cells involved in antitumor immunity than in the presence of Nrf2. Therefore, Nrf2 suppresses tumor cell development in the microenvironment. This function of Nrf2 in myeloid cells is in contrast to the phenomena in tumor cells, in which Nrf2-activation has been widely recognized to support tumor cell survival.

In chemical carcinogenesis experiments, Nrf2 has been considered to encourage cancer chemoprevention, and Nrf2 appears to be the key tumor-preventing transcription factor. In fact, Nrf2 detoxifies ROS and cytotoxic electrophiles that cause DNA damage. In some chemical carcinogenesis models, Nrf2 deficiency has been shown to increase the frequency of tumor occurrence [54–56].

However, somatic mutations in Keap1 and Nrf2 that interrupt Keap1-Nrf2 association and lead to constitutive Nrf2 activation are frequently detected in human cancers [57–59]. The latter observation indicates that Nrf2 activation is beneficial to the selfish growth of cancer cells. An important recent discovery is that this tumor-supporting effect of Nrf2 is mediated by not only the enhancement of cellular protection from stress but also the redirection of metabolic pathways to nucleotide synthesis in support of rapid cellular proliferation [29]. Taken together, Nrf2 protects normal cells from tumorigenesis but also helps the growth and survival of already developed tumors.

By contrast, Nrf2 expression in myeloid cells is required for repressing tumors. In myeloid cells surrounding tumors, Nrf2 eliminates ROS in MDSCs that attenuate antitumor immunity [52]. Metastasis experiments using the intravenous injection of Lewis lung carcinoma (3LL) cells clearly indicate that Nrf2 deficiency increases the metastasis of 3LL cells to the lung. Consistent with this observation, Nrf2 activation by Keap1 knockdown results in a reduction in the metastasis of 3LL cells; this observation was reproducible in a second cell line, melanoma-derived B16-F10. Bone marrow transplantation has revealed that the proper expression and function of Nrf2 are required in myeloid cells to suppress the metastasis of 3LL cells.

In tumor-bearing Nrf2-deficient mice, both the number and the ROS levels of MDSCs are increased. The putative mechanisms for the tumor suppressive activity of MDSCs depend on diverse mediators including NO, peroxynitrite produced from NO and superoxide anion, and ROS [53]. As intracellular ROS levels are elevated in Nrf2-deficient MDSCs, we hypothesize that the Nrf2 deficiency activates MDSCs possibly through an increase in ROS accumulation, resulting in the repression of antitumor immunity and an enhancement of metastasis. The demonstration of this link remains to be established.

## 7. Atherosclerosis and Nrf2

In contrast to many other inflammatory disorders alleviated by Nrf2, atherosclerosis is exacerbated by Nrf2. However, the molecular basis of this phenomenon has yet to be elucidated. In apolipoprotein E- (ApoE-) deficient atherosclerotic mice, multiple investigators have revealed that Nrf2 deficiency reduces atherosclerotic lesions in both high-fat diet (HFD)- and normal chow-fed ApoE-deficient mice [60–63] (Figure 4). No apparent change is found in the blood glucose levels, plasma lipid levels, or body weights of these mice, except that HFD-fed ApoE-deficient mice show increaces in serum triglycerides and glucose caused by the Nrf2 deficiency [60]. Similar to the conventional genetic deletion of Nrf2, the transplantation of Nrf2-deficient bone marrow cells reduces atherosclerotic lesions in ApoE-deficient recipient mice, indicating that Nrf2 induction in myeloid cells is proatherogenic [63].

The Nrf2-mediated exacerbation of atherosclerosis may be attributable to the upregulation of *CD36* expression by Nrf2 [64]. CD36 is a scavenger receptor required for the uptake of oxidized lipids by macrophages; thus, the increase in *CD36* expression is expected to promote foam cell transformation and atherosclerotic plaque formation. Nrf2 activates *CD36* gene expression by binding to an ARE located upstream of exon 1A1 [65]. In peritoneal macrophages, *CD36* expression is activated in response to oxidized low-density lipoprotein (LDL) in an Nrf2-dependent manner [64]. In *ApoE* and *Nrf2* double-knockout mice, *CD36* expression is downregulated under both HFD- and normal chow-fed conditions. Consequently, these mice are less atherosclerotic than *ApoE* single-knockout mice [60, 61]. These observations suggest that CD36 downregulation by Nrf2 deficiency prevents atherosclerosis in these mice.

Consistent with this hypothesis, the reduced uptake of oxidized LDL has been observed in Nrf2-deficient peritoneal macrophages compared with wild-type macrophages [61, 64]. However, this point is controversial, as similar levels of oxidized LDL uptake were observed between Nrf2-deficient and wild-type macrophages in a different study [62]. Furthermore, in another study, an increased uptake of modified LDLs was observed in Nrf2-deficient macrophages [66]. Thus, the mechanisms underlying the association between *CD36* expression and modified LDL uptake by macrophages remain unknown.

An alternative mechanism has been suggested for the proatherogenic function of Nrf2. Cholesterol crystals induce the production of the proinflammatory cytokines IL-1*α* and IL-1*β* in the presence of Nrf2 but not in Nrf2-deficient macrophages [62]. Importantly, the improvement of atherosclerosis in Nrf2-deficient mice disappeared when IL-1*α* and IL-1*β* were neutralized by antibodies. These results support the contention that these cytokines are important for mediating the proatherogenic function of Nrf2.

It is interesting to note that in clear contrast to the situation in ApoE-deficient mice, LDL receptor (Ldlr) and Nrf2 double-deficient mice have exacerbated atherosclerotic phenotypes compared with Ldlr single-knockout mice. The bone marrow transplantation of Nrf2-deficient cells into HFD-fed Ldlr-deficient mice exacerbates atherosclerosis compared with the transplantation of wild-type cells [66, 67]. These observations indicate that Nrf2 is anti-atherogenic in myeloid-derived cells of Ldlr-deficient mice.

Nrf2 deficiency in peritoneal macrophages upregulates the expression of two receptors, scavenger receptor A and lectin-like oxidized LDL receptor-1, that contribute to the uptake of modified lipids in response to oxidized lipids [66]. Similarly, lipid uptake and *Il6* expression are also upregulated in Nrf2-deficient peritoneal macrophages compared with wild-type macrophages [66]. Collectively, Nrf2 appears to play anti-atherogenic roles in Ldlr-deficient myeloid cells. However, because of the complexity of the atherogenic processes, which involves neutrophil infiltration, lipid uptake, inflammation and death of macrophages, a mechanistic understanding of how Nrf2 affects atherogenic processes remains elusive. Nonetheless, currently available data demonstrate that Nrf2 is definitely involved in the control of atherosclerosis.

## 8. Concluding Remarks

The Keap1-Nrf2 system is essential for protecting animals from environmental stresses. In myeloid-derived cells, phagocytosis and inflammatory regulation appear to be regulated by the Keap1-Nrf2 system. Nrf2 deficiency influences various functions of myeloid cells, which protect animals through anti-inflammatory and antitumor immunity activities. The functions of Nrf2 in both the progression of and protection against atherosclerosis remain elusive, and further studies are important.

## References

[1] Itoh K, Chiba T, Takahashi S (1997). An Nrf2/small Maf heterodimer mediates the induction of phase II detoxifying enzyme genes through antioxidant response elements. *Biochemical and Biophysical Research Communications*.

[2] Taguchi K, Motohashi H, Yamamoto M (2011). Molecular mechanisms of the Keap1-Nrf2 pathway in stress response and cancer evolution. *Genes to Cells*.

[3] Itoh K, Wakabayashi N, Katoh Y, Ishii T, O’Connor T, Yamamoto M (2003). Keap1 regulates both cytoplasmic-nuclear shuttling and degradation of Nrf2 in response to electrophiles. *Genes to Cells*.

[4] McMahon M, Itoh K, Yamamoto M, Hayes JD (2003). Keap1-dependent proteasomal degradation of transcription factor Nrf2 contributes to the negative regulation of antioxidant response element-driven gene expression. *The Journal of Biological Chemistry*.

[5] Kobayashi A, Kang MI, Okawa H (2004). Oxidative stress sensor Keap1 functions as an adaptor for Cul3-based E3 ligase to regulate proteasomal degradation of Nrf2. *Molecular and Cellular Biology*.

[6] Kobayashi A, Kang MI, Watai Y (2006). Oxidative and electrophilic stresses activate Nrf2 through inhibition of ubiquitination activity of Keap1. *Molecular and Cellular Biology*.

[7] Friling RS, Bensimon A, Tichauer Y, Daniel V (1990). Xenobiotic-inducible expression of murine glutathione S-transferase Ya subunit gene is controlled by an electrophile-responsive element. *Proceedings of the National Academy of Sciences of the United States of America*.

[8] Rushmore TH, Morton MR, Pickett CB (1991). The antioxidant responsive element: activation by oxidative stress and identification of the DNA consensus sequence required for functional activity. *The Journal of Biological Chemistry*.

[9] Kobayashi M, Yamamoto M (2005). Molecular mechanisms activating the Nrf2-Keap1 pathway of antioxidant gene regulation. *Antioxidants and Redox Signaling*.

[10] Dinkova-Kostova AT, Holtzclaw WD, Cole RN (2002). Direct evidence that sulfhydryl groups of Keap1 are the sensors regulating induction of phase 2 enzymes that protect against carcinogens and oxidants. *Proceedings of the National Academy of Sciences of the United States of America*.

[11] Itoh K, Wakabayashi N, Katoh Y (1999). Keap1 represses nuclear activation of antioxidant responsive elements by Nrf2 through binding to the amino-terminal Neh2 domain. *Genes and Development*.

[12] Zhang DD, Lo SC, Cross JV, Templeton DJ, Hannink M (2004). Keap1 is a redox-regulated substrate adaptor protein for a Cul3-dependent ubiquitin ligase complex. *Molecular and Cellular Biology*.

[13] Holtzclaw WD, Dinkova-Kostova AT, Talalay P (2004). Protection against electrophile and oxidative stress by induction of phase 2 genes: the quest for the elusive sensor that responds to inducers. *Advances in Enzyme Regulation*.

[14] Itoh K, Mochizuki M, Ishii Y (2004). Transcription factor Nrf2 regulates inflammation by mediating the effect of 15-deoxy-Δ^12, 14^-prostaglandin J_2_. *Molecular and Cellular Biology*.

[15] McMahon M, Lamont DJ, Beattie KA, Hayes JD (2010). Keap1 perceives stress via three sensors for the endogenous signaling molecules nitric oxide, zinc, and alkenals. *Proceedings of the National Academy of Sciences of the United States of America*.

[16] Um HC, Jang JH, Kim DH (2011). Nitric oxide activates Nrf2 through S-nitrosylation of Keap1 in PC12 cells. *Nitric Oxide*.

[17] Sawa T, Zaki MH, Okamoto T (2007). Protein S-guanylation by the biological signal 8-nitroguanosine 3',5'-cyclic monophosphate. *Nature Chemical Biology*.

[18] Fujii S, Sawa T, Ihara H (2010). The critical role of nitric oxide signaling, via protein S-guanylation and nitrated cyclic GMP, in the antioxidant adaptive response. *The Journal of Biological Chemistry*.

[19] Tsujita T, Li L, Nakajima H (2011). Nitro-fatty acids and cyclopentenone prostaglandins share strategies to activate the Keap1-Nrf2 system: a study using green fluorescent protein transgenic zebrafish. *Genes to Cells*.

[20] Kansanen E, Bonacci G, Schopfer FJ (2011). Electrophilic nitro-fatty acids activate Nrf2 by a Keap1 cysteine 151-independent mechanism. *The Journal of Biological Chemistry*.

[21] Ishii T, Itoh K, Takahashi S (2000). Transcription factor Nrf2 coordinately regulates a group of oxidative stress-inducible genes in macrophages. *The Journal of Biological Chemistry*.

[22] Kwak MK, Wakabayashi N, Itoh K, Motohashi H, Yamamoto M, Kensler TW (2003). Modulation of gene expression by cancer chemopreventive dithiolethiones through the Keap1-Nrf2 pathway: identification of novel gene clusters for cell survival. *The Journal of Biological Chemistry*.

[23] Hayes JD, Chanas SA, Henderson CJ (2000). The Nrf2 transcription factor contributes both to the basal expression of glutathione S-transferases in mouse liver and to their induction by the chemopreventive synthetic antioxidants, butylated hydroxyanisole and ethoxyquin. *Biochemical Society Transactions*.

[24] Banning A, Deubel S, Kluth D, Zhou Z, Brigelius-Flohé R (2005). The GI-GPx gene is a target for Nrf2. *Molecular and Cellular Biology*.

[25] Wild AC, Moinova HR, Mulcahy RT (1999). Regulation of *γ*-glutamylcysteine synthetase subunit gene expression by the transcription factor Nrf2. *The Journal of Biological Chemistry*.

[26] Alam J, Stewart D, Touchard C, Boinapally S, Choi AMK, Cook JL (1999). Nrf2, a Cap’n’Collar transcription factor, regulates induction of the heme oxygenase-1 gene. *The Journal of Biological Chemistry*.

[27] Aoki Y, Sato H, Nishimura N, Takahashi S, Itoh K, Yamamoto M (2001). Accelerated DNA adduct formation in the lung of the Nrf2 knockout mouse exposed to diesel exhaust. *Toxicology and Applied Pharmacology*.

[28] Enomoto A, Itoh K, Nagayoshi E (2001). High sensitivity of Nrf2 knockout mice to acetaminophen hepatotoxicity associated with decreased expression of ARE-regulated drug metabolizing enzymes and antioxidant genes. *Toxicological Sciences*.

[29] Mitsuishi Y, Taguchi K, Kawatani Y (2012). Nrf2 redirects glucose and glutamine into anabolic pathways in metabolic reprogramming. *Cancer Cell*.

[30] Komatsu M, Kurokawa H, Waguri S (2010). The selective autophagy substrate p62 activates the stress responsive transcription factor Nrf2 through inactivation of Keap1. *Nature Cell Biology*.

[31] Jain A, Lamark T, Sjøttem E (2010). *p62/SQSTM1* is a target gene for transcription factor NRF2 and creates a positive feedback loop by inducing antioxidant response element-driven gene transcription. *The Journal of Biological Chemistry*.

[32] Hoetzenecker W, Echtenacher B, Guenova E (2011). ROS-induced ATF3 causes susceptibility to secondary infections during sepsis-associated immunosuppression. *Nature Medicine*.

[33] Kobayashi M, Li L, Iwamoto N (2009). The antioxidant defense system Keap1-Nrf2 comprises a multiple sensing mechanism for responding to a wide range of chemical compounds. *Molecular and Cellular Biology*.

[34] Takaya K, Suzuki T, Motohashi H (2012). Validation of the multiple sensor mechanism of the Keap1-Nrf2 system. *Free Radical Biology and Medicine*.

[35] Groeger AL, Cipollina C, Cole MP (2010). Cyclooxygenase-2 generates anti-inflammatory mediators from omega-3 fatty acids. *Nature Chemical Biology*.

[36] Schreck R, Rieber P, Baeuerle PA (1991). Reactive oxygen intermediates as apparently widely used messengers in the activation of the NF-*κ*B transcription factor and HIV-1. *The EMBO Journal*.

[37] Gloire G, Legrand-Poels S, Piette J (2006). NF-*κ*B activation by reactive oxygen species: fifteen years later. *Biochemical Pharmacology*.

[38] Powers KA, Szászi K, Khadaroo RG (2006). Oxidative stress generated by hemorrhagic shock recruits Toll-like receptor 4 to the plasma membrane in macrophages. *Journal of Experimental Medicine*.

[39] Nakahira K, Kim HP, Geng XH (2006). Carbon monoxide differentially inhibits TLR signaling pathways by regulating ROS-induced trafficking of TLRs to lipid rafts. *Journal of Experimental Medicine*.

[40] Kong X, Thimmulappa R, Kombairaju P, Biswal S (2010). NADPH oxidase-dependent reactive oxygen species mediate amplified TLR4 signaling and sepsis-induced mortality in Nrf2-deficient mice. *Journal of Immunology*.

[41] Kong X, Thimmulappa R, Craciun F (2011). Enhancing Nrf2 pathway by disruption of Keap1 in myeloid leukocytes protects against sepsis. *American Journal of Respiratory and Critical Care Medicine*.

[42] Harvey CJ, Thimmulappa RK, Sethi S (2011). Targeting Nrf2 signaling improves bacterial clearance by alveolar macrophages in patients with COPD and in a mouse model. *Science Translational Medicine*.

[43] Hou Y, Xue P, Bai Y (2012). Nuclear factor erythroid-derived factor 2-related factor 2 regulates transcription of CCAAT/enhancer-binding protein *β* during adipogenesis. *Free Radical Biology and Medicine*.

[44] Huber R, Pietsch D, Panterodt T, Brand K (2012). Regulation of C/EBP*β* and resulting functions in cells of the monocytic lineage. *Cellular Signaling*.

[45] Iizuka T, Ishii Y, Itoh K (2005). Nrf2-deficient mice are highly susceptible to cigarette smoke-induced emphysema. *Genes to Cells*.

[46] Cho HY, Jedlicka AE, Reddy SPM (2002). Role of NRF2 in protection against hyperoxic lung injury in mice. *American Journal of Respiratory Cell and Molecular Biology*.

[47] Reddy NM, Kleeberger SR, Kensler TW, Yamamoto M, Hassoun PM, Reddy SP (2009). Disruption of Nrf2 impairs the resolution of hyperoxia-induced acute lung injury and inflammation in mice. *Journal of Immunology*.

[48] Athale J, Ulrich A, MacGarvey NC (2012). Nrf2 promotes alveolar mitochondrial biogenesis and resolution of lung injury in *Staphylococcus aureus* pneumonia in mice. *Free Radical Biology and Medicine*.

[49] Rangasamy T, Guo J, Mitzner WA (2005). Disruption of Nrf2 enhances susceptibility to severe airway inflammation and asthma in mice. *Journal of Experimental Medicine*.

[50] Thimmulappa RK, Lee H, Rangasamy T (2006). Nrf2 is a critical regulator of the innate immune response and survival during experimental sepsis. *Journal of Clinical Investigation*.

[51] Ishii Y, Itoh K, Morishima Y (2005). Transcription factor Nrf2 plays a pivotal role in protection against elastase-induced pulmonary inflammation and emphysema. *Journal of Immunology*.

[52] Satoh H, Moriguchi T, Taguchi K (2010). Nrf2-deficiency creates a responsive microenvironment for metastasis to the lung. *Carcinogenesis*.

[53] Gabrilovich DI, Nagaraj S (2009). Myeloid-derived suppressor cells as regulators of the immune system. *Nature Reviews Immunology*.

[54] Ramos-Gomez M, Kwak MK, Dolan PM (2001). Sensitivity to carcinogenesis is increased and chemoprotective efficacy of enzyme inducers is lost in *nrf2* transcription factor-deficient mice. *Proceedings of the National Academy of Sciences of the United States of America*.

[55] Iida K, Itoh K, Kumagai Y (2004). Nrf2 is essential for the chemopreventive efficacy of oltipraz against urinary bladder carcinogenesis. *Cancer Research*.

[56] Ohkoshi A, Suzuki T, Ono M (2013). Roles of Keap1-Nrf2 system in upper aerodigestive tract carcinogenesis. *Cancer Prevention Research*.

[57] Shibata T, Ohta T, Tong KI (2008). Cancer related mutations in NRF2 impair its recognition by Keap1-Cul3 E3 ligase and promote malignancy. *Proceedings of the National Academy of Sciences of the United States of America*.

[58] Ohta T, Iijima K, Miyamoto M (2008). Loss of Keap1 function activates Nrf2 and provides advantages for lung cancer cell growth. *Cancer Research*.

[59] Guichard C, Amaddeo G, Imbeaud S (2012). Integrated analysis of somatic mutations and focal copy-number changes identifies key genes and pathways in hepatocellular carcinoma. *Nature Genetics*.

[60] Sussan TE, Jun J, Thimmulappa R (2008). Disruption of Nrf2, a key inducer of antioxidant defenses, attenuates ApoE-mediated atherosclerosis in mice. *PLoS ONE*.

[61] Barajas B, Che N, Yin F (2011). NF-E2-related factor 2 promotes atherosclerosis by effects on plasma lipoproteins and cholesterol transport that overshadow antioxidant protection. *Arteriosclerosis, Thrombosis, and Vascular Biology*.

[62] Freigang S, Ampenberger F, Spohn G (2011). Nrf2 is essential for cholesterol crystal-induced inflammasome activation and exacerbation of atherosclerosis. *European Journal of Immunology*.

[63] Harada N, Ito K, Hosoya T (2012). Nrf2 in bone marrow-derived cells positively contributes to the advanced stage of atherosclerotic plaque formation. *Free Radical Biology and Medicine*.

[64] Ishii T, Itoh K, Ruiz E (2004). Role of Nrf2 in the regulation of CD36 and stress protein expression in murine macrophages: activation by oxidatively modified LDL and 4-hydroxynonenal. *Circulation Research*.

[65] Maruyama A, Tsukamoto S, Nishikawa K (2008). Nrf2 regulates the alternative first exons of *CD36* in macrophages through specific antioxidant response elements. *Archives of Biochemistry and Biophysics*.

[66] Ruotsalainen AK, Inkala M, Partanen ME (2013). The absence of macrophage Nrf2 promotes early atherogenesis. *Cardiovascular Research*.

[67] Collins AR, Gupte AA, Ji R (2012). Myeloid deletion of nuclear factor erythroid 2-related factor 2 increases atherosclerosis and liver injury. *Arteriosclerosis, Thrombosis, and Vascular Biology*.

